# Recent Advances in the Application of Natural Products for Postharvest Edible Mushroom Quality Preservation

**DOI:** 10.3390/foods13152378

**Published:** 2024-07-27

**Authors:** Yuxin Liufang, Yi Wu, Huabin Zhou, Hang Qu, Hailong Yang

**Affiliations:** 1 College of Life & Environmental Science, Wenzhou University, Wenzhou 325035, China; lfyx0301@163.com (Y.L.); 21211231224@stu.wzu.edu.cn (Y.W.); zhb@wzu.edu.cn (H.Z.); 2 Zhejiang Provincial Key Laboratory for Water Environment and Marine Biological Resources Protection, Wenzhou University, Wenzhou 325035, China

**Keywords:** edible mushrooms, natural products, postharvest, preservation, quality deterioration, storage

## Abstract

Edible mushrooms are favored by consumers for their excellent nutritional value and pharmacological properties. However, fresh mushrooms are highly perishable and undergo rapid quality deterioration induced by a series of intrinsic and extrinsic factors during postharvest storage. In recent years, the application of natural products derived from plants, animals, microorganisms, and other sources in mushroom quality preservation has drawn increasing attention. Compared to chemical preservatives, natural products show similar or higher biological activity and have few side effects on human health. This review summarizes the recent advances in the application of natural products used for quality maintenance of postharvest mushrooms. These natural substances mainly include essential oils, polyphenols, polysaccharides, bacteriocins, and other extracts. They have the potential to inhibit mushroom weight loss, softening, and browning, reduce the count of pathogenic microorganisms, and retain nutrients and flavor, effectively improving the quality of mushrooms and extending their shelf-life. The preservation techniques for natural products and their preservation mechanisms are also discussed here. Overall, this review provides current knowledge about natural products in edible mushroom preservation and aims to inspire more in-depth theoretical research and promote further practical application.

## 1. Introduction

Mushrooms are macro-fungi of the phylum Basidiomycota with distinctive fruiting bodies, which have been consumed as a traditional food for centuries. There are 3000 edible mushroom species in nature, but only approximately 35 species are commercially cultivated and widely accepted as food products [1]. Among them, *Agaricus bisporus* (button mushroom) is the most extensively cultivated and consumed globally, followed by *Lentinula edodes* (shiitake mushroom) and *Pleurotus eryngii* (oyster mushroom) [2]. Edible mushrooms are well acknowledged as a valuable diet and are usually called the “meat for the poor” due to their excellent nutrition and relatively inexpensive price. They are rich in proteins, amino acids, and dietary fibers, as well as low in fats and calories, catering to the advocated nutritious dietary concept [3]. Additionally, they are fine sources of vitamins (thiamine, riboflavin, vitamin B12, etc.) and essential minerals (iron, magnesium, phosphorus, etc.) [4]. In addition, edible mushrooms contain diverse bioactive components such as polysaccharides, polyphenols, peptides, terpenoids, and so on, which contribute to potential health benefits, including anti-diabetic, anti-tumor, antioxidant, anti-inflammatory, anti-cholesterolemic, anti-hypertensive, neuroprotective, immunomodulatory, and hepatoprotective activities [5,6]. Owing to their outstanding nutritional value and pharmacological properties, edible mushrooms have become increasingly attractive to consumers and have been desirably incorporated into daily meals. As a result, the global consumption of edible mushrooms has increased annually by 7% in recent years. Based on data from the Food and Agriculture Organization Statistical (FAOSTAT), total mushroom production is expected to keep increasing and reach 20.84 million tons by the year 2026 [7].

However, fresh edible mushrooms are highly perishable and not suitable for prolonged storage or long-distance transportation [8]. Continuous postharvest quality deterioration poses a challenge to the commercialization and future development of edible mushrooms. The quality changes of mushrooms mainly focus on weight loss, browning, softening, nutrient loss, and flavor loss [9]. Moisture retention is a critical factor in mushroom freshness. Moisture migration and loss over a short period of time cause weight loss and texture changes [10]. After harvesting, mushrooms still exhibit intense respiration and high enzymatic activity, leading to the rapid degradation of nutrients, elongation of stipes, cap opening, browning, and even autolysis [2]. Moreover, due to their lack of a protective epidermal structure, mushrooms are vulnerable to mechanical damage and microbial spoilage during postharvest storage and transportation. All these internal and external factors collectively contribute to the quality loss of edible mushrooms. These undesirable changes in appearance and texture make them unacceptable to consumers, ultimately resulting in a significant decline in economic value. Therefore, it is urgent and necessary to adopt a proper strategy to maintain the quality of edible mushrooms and extend their shelf-life.

To date, a variety of approaches have been developed and applied to maintain mushroom quality, including thermal, physical, and chemical processing [9]. The addition of preservatives is the most widely used preservation method and has the advantages of convenience, low price, and remarkable effects. In the recent years, chemical synthetic preservatives have been gradually abandoned due to their undesirable residues, potential allergens, and possible formation of carcinogenic compounds [11]. In contrast, natural products are usually rich in bioactive compounds, show similar or even higher biological activity, and have few side effects with respect to human health [12]. Thus, an increasing number of natural products are being utilized to retard the quality degradation of postharvest fruits and vegetables [13,14]. The effective combination of natural products with appropriate preservation technology is conducive to maintaining postharvest quality and extending the shelf-life of edible mushrooms, which is beneficial to the development of the industry as well as global consumers [15].

Despite the importance of natural products in edible mushroom preservation, no recent review is available to provide the research advances in this field. Therefore, this paper aims to survey the published papers from the past ten years and provide an overview of the application of natural products in maintaining mushroom quality. The preservation techniques for natural products and their preservation mechanisms are also discussed. It is anticipated that this review will inspire theoretical research and encourage the practical application of more natural products in food preservation.

## 2. Quality Deterioration of Postharvest Edible Mushrooms

**Figure 1:**
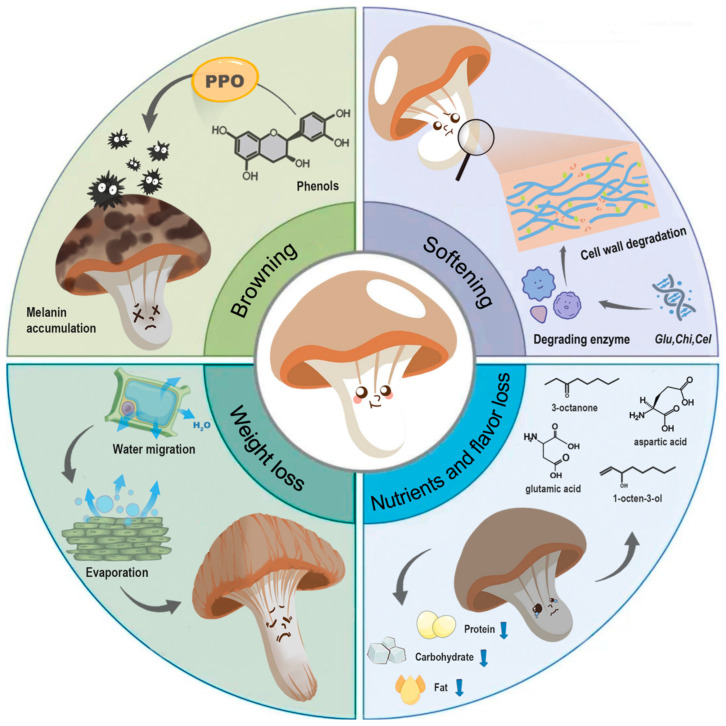
Changes in quality of postharvest edible mushrooms, including weight loss, browning, softening, and loss of nutrients and flavor.

Edible mushrooms are highly perishable products and only have 1~3 days of shelf-life at ambient temperatures. After harvesting, mushrooms undergo rapid senescence due to a combination of intrinsic and extrinsic factors. These changes are primarily characterized by weight loss, browning, softening, and loss of nutrients and flavor (Figure 1), significantly impacting the quality of the mushrooms and reducing their value in the market.

### 2.1. Weight Loss

The weight loss of edible mushrooms is predominantly due to moisture loss. Water, which makes up 85% to 95% of fresh mushrooms, is mainly stored in compartments differentiated by cellular structure, and is responsible for cellular integrity [16]. Therefore, moisture retention is a crucial factor in determining mushroom freshness. During postharvest storage, water migrates from cells or extracellular spaces towards the external surface and then evaporates. Consequently, the moisture content gradually decreases, leading to overall weight loss and tissue softening [10]. The disruption of cell wall and cell membrane structures can diminish the water holding capacity of mushroom cells, exacerbating moisture loss and weight loss [9]. Any weight loss exceeding 5% of the fresh mass results in quality deterioration, rending the mushrooms unsuitable for commercial sale [17]. The rate of moisture loss primarily hinges on the maturity of mushrooms and their storage conditions (temperature, relative humidity, air circulation, atmospheric pressure, etc.). To maintain the freshness of edible mushrooms, it is essential to control moisture loss at a relatively low level during postharvest storage.

### 2.2. Browning

Surface color is the most obvious quality attribute of edible mushrooms. During prolonged storage, mushrooms tend to acquire brown spots. This phenomenon is referred to as browning, and seriously affects the overall appearance of mushrooms and changes consumers’ purchasing behavior [17]. Enzymatic reactions are considered the predominant cause of browning, with polyphenol oxidase (PPO) being the key enzyme. In this process, phenolic substances are catalyzed into quinones, which are then oxidized to melanin, resulting in undesired browning. The amount of PPO, activation of latent enzyme forms, and contents and types of phenolic substrates, as well as the access of substrates to the active enzymes, are all factors influencing the enzymatic browning process of postharvest mushrooms [18]. During postharvest transportation and storage periods, browning can be induced by various factors, such as mechanical damage, temperature, relative humidity, respiration, and microbial contamination. Direct mechanical damage causes the breakdown of cell membranes, allowing contact between PPO, phenolic substrates, and oxygen, thereby causing browning within a short period of time [19]. High temperature, low relative humidity, and intense respiration tend to enhance the activity of PPO and accelerate discoloration. Additionally, mushrooms are highly sensitive to pathogens, which are also considered potential factors causing browning. Their secreted toxins, such as tolaasin, have been found to be involved in the discoloration of edible mushrooms [20].

### 2.3. Softening

Firmness is one of the textural characteristics that represent the degree of softening of edible mushrooms. During postharvest storage, firmness decreases rapidly, leading to a shortened shelf-life and an increased risk of microbial contamination [9]. The decline in firmness predominantly results from the degradation of cell wall components and structural changes. Unlike other fruits and vegetables, the skeletal structures of mushroom cell walls are primarily composed of glucan, chitin, and cellulose [21]. Glucan, accounting for 80–90% of dry cell wall weight, is located on the cell surface and plays a crucial role in connecting cells, assembling chitin, and forming cell wall scaffolds. Chitin, a relatively minor but structurally important component, is mainly responsible for creating the rigid microfibril structure, directly influencing cell wall strength. Generally, the contents of these components decrease with prolonged storage duration, and the trend is consistent with that of firmness [22]. Cell wall-degrading enzymes are considered the major cause of changes in cell wall components. Many enzymes and genes related to the disassembly of cell wall components have been identified as being involved in mushroom softening, such as endo-*β*-1,3-glucanase (encoded by *Leglu1* and *Letlg1*), exo-*β*-1,3-glucanase (encoded by *Leexg1* and *Leexg2*), chitinase (encoded by *Chi1* and *Chi2*), and cellulase (encoded by *Cel1*) [22,23,24]. During storage, factors such as mechanical damage, microbial attack, and thermal treatment up-regulate the related genes and enhance the activities of degrading enzymes, thereby exacerbating mushroom softening [2].

### 2.4. Nutrients and Flavor Loss

Edible mushrooms are rich in carbohydrates, fiber, and protein and low in fat. For instance, dried button mushrooms contain 74% carbohydrates, 14.1% protein, 2.2% fat, and 9.7% ash [25]. Carbohydrates and proteins are the primary nutrients that support the metabolism of postharvest mushrooms [26]. A decrease in sugars and proteins is a crucial indicator of mushroom quality deterioration. Typically, the contents of total sugar, soluble proteins, and fat gradually decrease over time during storage, while the level of reducing sugar increases due to the hydrolysis of macro-molecular carbohydrates. The loss of nutrients significantly affects the nutritional value of edible mushrooms. Additionally, mushrooms have a unique aroma and flavor attributed to their volatile components (alcohols, sulfur compounds, aldehydes, ketones, acids, esters, etc.) and non-volatile components (soluble sugars, free amino acids, organic acids, nucleotides, etc.). Research indicates that many species of fresh mushrooms have distinct flavors such as 1-octen-3-ol, 3-octanone, and 1-octanol [27]. Umani 5′-nucleotides and amino acids are the main nonvolatile components, including adenosine/guanosine/inosine-5′-monophosphate, glutamic acid, and aspartic acid [28]. Prolonged storage can lead to flavor loss, with a noticeable decline in these characteristic substances [29]. However, the mechanism of mushroom flavor loss remains speculative, and few related genes have been identified or studied [17].

## 3. Natural Products Used for Edible Mushroom Preservation

**Figure 2:**
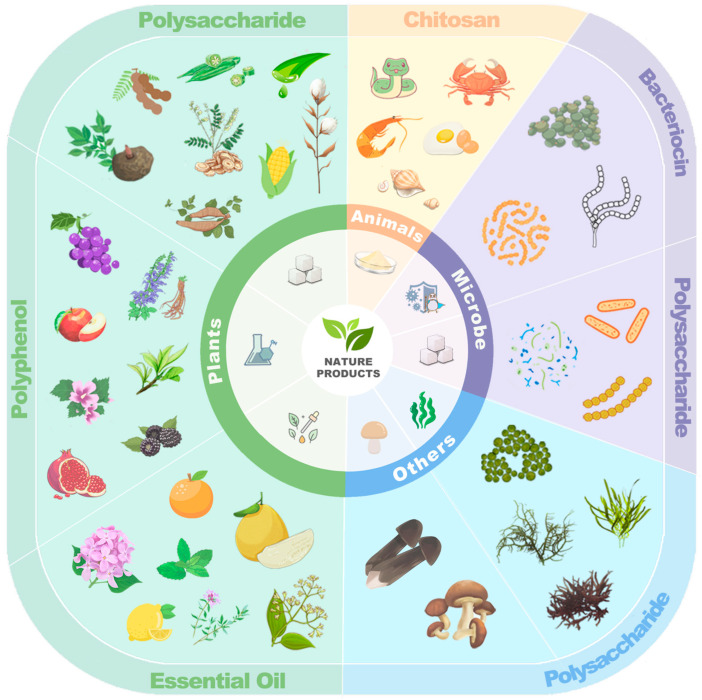
Classification of natural products utilized in edible mushroom preservation.

In recent years, there has been a significant increase in the interest surrounding the use of natural products as preservatives for preserving the quality of postharvest fruits and vegetables. A wide variety of natural products derived from plants, animals, microbes, and other sources have demonstrated the potential to effectively prolong the quality of edible mushrooms, as illustrated in Figure 2.

### 3.1. Plant-Derived Extracts

#### 3.1.1. Essential Oils

Essential oils (EOs) are natural, volatile, and aromatic liquids extracted from various plant parts, such as trunks, leaves, flowers, fruits etc.). They are known for being safe, cost-effective, easily biodegradable, and environmentally friendly [30]. The significant antioxidant and antimicrobial properties of EOs make them excellent alternatives to synthetic preservatives [31]. The use of EOs in food preservation has been widely recognized [32]. An increasing amount of research has emphasized the effectiveness of EOs in maintaining the quality of edible mushrooms.

In earlier years, fumigation with clove, cinnamaldehyde, and thyme oils was found to effectively maintain the sensory quality of mushrooms [15,33]. Subsequently, peppermint, lemongrass, citronella, and mint oils have also been reported by Qu et al. [34] and Manjari et al. [35]. These EOs evidently delayed mushroom browning, softening, and cap opening, as well as promoted the accumulation of phenolics, ascorbic acid, soluble proteins, and total sugars [15,34]. Moreover, Jiang et al. indicated that EOs significantly improved the antioxidant capacity of postharvest mushrooms [33]. The activities of catalase (CAT), superoxide dismutase (SOD), ascorbate peroxidase (APX), and glutathione reductase (GR) in shiitake mushrooms were significantly enhanced by cinnamaldehyde oil. Further, the activities of PPO and peroxidase (POD) in oyster mushrooms were markedly inhibited by mint oil [35]. In essence, these enzymes are regulated by related genes. For instance, peppermint oil affected the expression of PPO and phenylalanine ammonia lyase (PAL) in button mushrooms mainly through regulating the genes of *AbPPO* and *AbPAL* [34].

However, the physical and chemical properties of EOs are not stable enough, making it difficult to maintain sustained release and achieve long-term effects. To enhance the stability and persistence of EOs, a controlled delivery system suitable for food applications is essential [36]. In recent years, significant efforts have been made to develop edible coatings and active packaging films containing EOs to broaden their utilization in food preservation. The most commonly used method involves adding EOs to active coatings/films to create an emulsion solution. During the emulsification process, an EO is evenly distributed in lipid droplets within a polymer matrix. Echegoyen et al. (2015) discovered that paper containing cinnamon EO based on a paraffin emulsion was more effective against weight loss and mushroom browning compared to paper based on solids [11]. Moreover, the emulsion formation masked the odor of EOs, enhanced their stability, and improved their controlled release. A tragacanth gum coating containing *Satureja khuzistanica* or *Zataria multiflora* Boiss. EOs also exhibited significant protection against the sensory quality degredation of button mushrooms, reducing respiration rate, cap opening, and discoloration [37]. A chitosan/zein complex film loaded with lemon EO has shown similar effects [38]. Additionally, Zhu et al. developed a controlled release packaging film using polylactic acid and mesoporous silica nanoparticles loaded with clove EO, effectively enhancing the postharvest quality of button mushrooms [39]. To prevent EO evaporation during the formation of edible coatings/active films, numerous studies have focused on maintaining the content of EOs in these coatings/films above a sufficient level [40]. Various promising approaches have been suggested for mushroom preservation, including encapsulation, nanofibers, nanoemulsion, Pickering emulsion, and multilayer systems. These strategies can enhance the stability and retention of EOs, thereby boosting their effectiveness.

Encapsulating EOs into edible coatings/active films can reduce the loss of EOs and withstand harsh conditions such as air, light, temperature, and humidity. For example, *Shao* et al. developed starch-based microcapsules loaded with cinnamon oil to extend the release time of the EO and delay the spoilage of button mushrooms [41]. Another common encapsulation method is through nanoparticles. Chitosan is frequently utilized as a raw material for producing nanoparticles. Karimirad et al. constructed chitosan nanoparticles containing *Citrus aurantium* EO, which effectively slowed down the quality degradation of mushrooms compared to fumigation [42]. Furthermore, chitosan nanoparticles loaded with EO enhanced the accumulation of phenolic compounds and ascorbic acid in mushrooms, along with boosting the activities of CAT and SOD. Cajuputi (*Melaleuca cajuputi* Powerll) EO was also successfully encapsulated into a chitosan nanomatrix, preserving the quality of coated button mushrooms [43].

Electrospinning is often utilized to produce nanofibers with high porosity and surface area [44]. To minimize the loss of EOs, Pan et al. prepared crosslinked electrospun polyvinyl alcohol/cinnamon EO/*β*-cyclodextrin nanofiber films for sustained release of antibacterial agents [45]. The nanofibers exhibited good antibacterial properties against *Staphylococcus aureus* and *Escherichia coli*, thereby delaying the decay of mushrooms during the postharvest storage period. Additionally, zein/ethyl cellulose hybrid nanofibers encapsulating cinnamon EO were employed for button mushroom preservation. Results indicated a significant improvement in the water resistance of the zein electrospun nanofibers, attributed to the hydrogen bonds between the hydroxyl groups of ethyl cellulose and the amino groups of zein. This active film demonstrated excellent efficacy in extending the shelf-life of button mushrooms [46]. Recently, Zhang et al. developed a novel cinnamon–clove compound EO microcapsules/graphene oxide/polyvinyl alcohol/polylactic acid composite film and investigated the effects of EO and nano-fillers on the postharvest quality of white beech mushrooms (*Hypsizygus marmoreus*). This active film showed strong antibacterial activity against *Mucor* and *Aspergillus niger*, significantly prolonging their shelf-life by 4 days at 4 °C [47].

Since most edible coatings/active films matrices are hydrophilic, coarse emulsions are typically produced using conventional methods. In comparison to coarse emulsions, nanoemulsions have higher stability and a larger surface area ratio, which reduces the explosive release resulting from the accumulation of EO itself and demonstrates improved performance in terms of bioavailability [48]. For example, an alginate-based coating containing a cinnamaldehyde oil nanoemulsion was constructed to preserve button mushrooms [49]. This nanoemulsion coating remarkably enhanced the antimicrobial properties, inhibited the senescence process, and extended the shelf-life of the mushrooms by reducing droplet size and improving uniform dispersion. Moosavi-Nasab et al. evaluated the impact of edible aloe vera and gelatin coatings incorporating a Shirazi thyme EO nanoemulsion on edible mushrooms and found that these coatings effectively inhibited the growth of microorganisms, including mesophilic bacteria, yeasts, and molds [50]. Additionally, Pickering emulsion has been utilized to strengthen the stability of EOs. Yang et al. developed a sodium alginate/guar gum-based nanocomposite film incorporating a *β*-Cyclodextrin/persimmon pectin-stabilized baobab seed oil Pickering emulsion [51]. They reported that this film acted as a barrier during postharvest storage, preventing the infiltration of water and oxygen, thereby contributing to the effective preservation of edible mushrooms.

In recent years, multilayer active films/coatings have attracted increasing attention for the preservation of edible mushrooms due to their exceptional mechanical and barrier properties. A composite bilayer film composed of corn starch and polylactic acid has been developed, featuring a hydrophobic outer layer and an absorbent inner layer. Eucalyptus leaf EO microcapsules were integrated into the inner layer, facilitating the release of the bioactive substances within the storage environment. This active film evidently suppressed the respiration rate, decreased the consumption of organic substances, and significantly extended the shelf-life of button mushrooms [52]. More recently, Feng et al. formulated an active film using glutenin and tamarind gum loaded with the binary microemulsion of melatonin and pummelo EO, offering advantages such as unidirectional sustained release, high barrier protection, and seamless adhesion between layers [53]. The outer barrier layer exerted excellent protective properties, minimizing external interference and the inefficient diffusion of EO within the inner layer. This active film notably improved the anti-oxidative properties of mushrooms and inhibited microbial growth during postharvest storage, thereby retarding their quality deterioration.

#### 3.1.2. Polyphenols

Polyphenols represent a significant group of secondary plant metabolites characterized by the presence of one or more aromatic rings with attached hydroxyl groups. Depending on their structural features, they can be categorized into flavonoids, phenolic acids, lignans, stilbenoids, coumarins, and tannin polymers [54]. The chemical structure of polyphenols determines their antioxidant capacity due to the availability of phenolic hydrogens as radical scavengers. The primary utilization of plant polyphenols in food preservation is linked to their antioxidative properties [55]. Furthermore, polyphenols exhibit antimicrobial characteristics and can function as inhibitors of pathogenic microorganisms [56].

Phenolic acids play a crucial role in edible mushroom preservation, with key examples being protocatechuic acid (PCA), gallic acid (GA), and caffeic acid (CA). PCA, a natural phenolic acid found in plants like *Salvia miltiorrhiza* and *Hibiscus*, has a broad antimicrobial spectrum and can inhibit tyrosinase. These properties make PCA a promising option for maintaining mushroom quality. For example, oyster mushrooms coated with PCA-grafted-chitosan solutions showed improved firmness and reduced weight loss, browning, respiration rate, electrolyte leakage, and levels of malondialdehyde (MDA), superoxide anion, and hydrogen peroxide [57]. Huang et al. prepared a PCA-CaCl_2_-NaCl-pullulan composite preservative for button mushrooms, resulting in enhanced appearance, antioxidant capacity, and preservation of nutrients and flavor compounds, extending their shelf-life to 12 days [29]. GA, another commonly used phenolic acid, exhibits potent antioxidant and antibacterial properties. In the storage test performed on fresh black truffles, GA drastically reduced the levels of *Pseudomonas*, *Enterobacteriaceae*, and *Eumycetes*. Additionally, the off-flavors were obviously absent and their shelf-life was extended to 28 days at 4 °C [58]. Packaging mushrooms with GA-grafted chitosan film or modified cellulose nanocrystal-grafted GA also significantly maintained their postharvest quality [59,60]. Furthermore, CA can help delay the deterioration of mushroom quality. Pei et al., developed a CA-grafted chitosan/polylactic acid film and investigated its effect on button mushrooms [61]. Compared to traditional polyethylene packaging, this film delayed browning and respiration rates, preserving the mushroom cells’ structure, likely due to the inhibition of phospholipid-degrading enzymes [61,62].

In addition to phenolic acids, several other natural plant-derived polyphenolic compounds have also been discovered in recent years. For instance, thymol has been found to maintain the hardness, color, total phenol content, cell membrane integrity, and total antioxidant capacity of button mushrooms, significantly extending their shelf-life [63,64]. Oxyresveratrol and resveratrol are natural polyphenol compounds and commonly extracted from grapes, mulberries, and other plants. Niu et al. investigated their effects on the quality of postharvest shiitake mushrooms. Oxyresveratrol outperformed resveratrol, significantly delaying browning and softening and reducing the occurrence of decay during storage. This protective effect is mainly attributed to inhibiting the oxidation and hydrolysis of phospholipids to mitigate the cellular damage of shiitake mushrooms [65].

The utilization of polyphenol-rich plant extracts, such as peel extract, seed extract, and tea extract, represents an alternate strategy that is currently the focus of extensive research [66]. These extracts, predominantly sourced from low-value byproducts or underutilized plant species, are gaining attention due to their potential economic value [67]. Grapefruit seed extract, historically used to inhibit microbial growth and preserve mushroom quality, is rich in flavonoids, phenolic acids, and other antimicrobial compounds [68]. Green tea extract, known for its catechin content, which are phenolic compounds with strong antioxidant and antimicrobial properties, has been studied for its effects on mushroom quality. Wrona et al. demonstrated a concentration of 0.6 g/m^2^ of green tea extract could maintain the white color of mushrooms longer compared to control samples [69]. Pistachio green hull, an inexpensive agricultural byproduct rich in phenolic compounds, has been found to suppress the enzymatic browning of button mushrooms as a potent natural tyrosinase inhibitor [70]. Pomegranates (*Punicagranatum* L.) peels, byproducts of pomegranate processing, are abundant in polyphenols, mainly in the forms of ellagitannins and ellagic acid. Lyn et al. successfully extended the shelf-life of oyster mushrooms from 3 days to 11 days at 4 °C by combining pomegranate peel extract with active packaging [71]. Similarly, the shelf-life of button mushrooms coated with apple peel powder, rich in phenolics, was effectively prolonged from 6 days to 9 days under refrigerated conditions [72].

#### 3.1.3. Polysaccharides

Polysaccharides are polymeric carbohydrate molecules composed of elongated chains of monosaccharide units connected by glycosidic bonds. Natural polysaccharides from plants have distinct properties attributed to their specific structural characteristics. Current research indicates that polysaccharides have significant potential in edible coatings/active packaging films to protect mushrooms against quality degradation, mainly including cellulose, starch, and natural gums [14].

Cellulose is an edible and biodegradable polymer that is widely distributed in the cell wall of plants. Various agricultural wastes, such as cotton stalks, fruits, vegetables, and forest residues, serve as abundant sources of cellulose. It is a linear polysaccharide composed of repeating units of *β*(1 → 4) linked D-glucose. The numerous intramolecular hydrogen bonds in cellulose chains contribute to its extended and stiff rod-like conformation. Due to its unique physical properties, cellulose and its derivatives are recognized as safe and environmentally friendly materials for food packaging. Cellulose can act as a carrier, readily encapsulating different active agents to preserve edible mushrooms [73]. Wang et al. extracted cellulose from byproducts of water bamboo (*Zizania latifolia*) to prepare polylactic acid/cellulose packaging for protecting the quality of shiitake mushrooms [74]. These cellulose-based biofilms loaded with cinnamaldehyde EO exhibited good performance in water resistance and effectively inhibited the mycelial growth and spore germination of *Aspergillus niger* and *Trichoderma harzianum* isolated from shiitake mushrooms. Cellulose nanocrystals (CNCs) are nanomaterials derived from natural sources like wood pulp, possessing high surface area, stiffness and strength. Modified CNCs incorporated into a gellan gum matrix markedly reduced the respiration rate and extended the shelf-life of mushrooms [60,75]. Similar outcomes of CNC bio-based films have also been documented by Louis et al. [76]. To improve their film-forming properties, cellulose derivatives are receiving increasing attention. For example, ethyl cellulose (EC), a widely used cellulose derivative, shows exceptional mechanical properties and strong water resistance. Zein/EC nanofibers combined with cinnamon EO effectively reduced weight loss and maintained the firmness of postharvest mushrooms [46]. Additionally, hydroxypropyl methylcellulose (HPMC), another cellulose derivative with high flexibility and insolubility, was utilized by Jiang et al. to create a composite biofilm with pueraria starch, significantly delaying the aging process of edible mushrooms, primarily by inhibiting respiration, ROS production, and discoloration [77].

Starch is highly susceptible to water and has a low water vapor barrier capacity due to its hydrophilic nature. The integration of starch-based polymers with other hydrophobic substances is commonly regarded as a versatile strategy for the development of materials with improved properties. Previously, Zhang et al. formulated a film using potato starch and mesoporous silica nanoparticles loaded with cinnamon EO to protect postharvest mushrooms from the CNRMA 03.0371 strain (*Mucor circinelloides*) and FJ09 species (*Mucor* sp.). This biofilm displayed excellent antimicrobial activity, particularly against FJ09 species [78]. Moreover, Guo et al. employed low-temperature plasma technology to modify potato starch, enhancing intermolecular interactions, and subsequently fabricated a modified potato starch-based film to preserve edible mushrooms. In the test, the packaged mushrooms exhibited enhanced appearance and retained significantly high activity of key enzymes in the phenylpropane metabolic pathway [79]. Additionally, corn starch is a suitable film-forming material, but resulting films often exhibit low tensile strength, poor transparency, and high hydrophilicity. To address these drawbacks, Chen et al. combined corn starch with polylactic acid to develop a composite biofilm for mushroom preservation and shelf-life extension [52]. Furthermore, pueraria starch has been utilized in biofilm preparation to delay the senescence of postharvest mushrooms [77].

Natural gums represent a category of cost-effective, non-toxic, and readily accessible polymeric polysaccharides. Among plant-derived gums are guar gum, pectin, arabic, karaya, tragacanth, ghatti, and kondagogu gum [80]. These gums can be utilized for producing coating/films with effective barrier properties against moisture and oxygen. For instance, tragacanth gum (TG), sourced from *Astragalus*, is a complex heterogeneous anionic branched polysaccharide with a high molecular weight. Due to its structural attributes, TG is commonly used as a carrier for active substances. Nasiri et al., incorporated *Zataria multiflora* Boiss. EO into TG to extend the shelf-life up of button mushrooms significantly, up to 16 days [8,81]. Konjac glucomannan (KGM) is a water-soluble macromolecular polysaccharide primarily composed of D-glucose and D-mannose linked by *β*-1,4-glycosidic bonds, exhibiting notable film-forming capacities. Zhang et al. developed a film using KGM and carrageenan (KC) supplemented with nano-silica to enhance mechanical property and permeability. This KGM/KC/nano-silica film substantially preserved the overall quality of mushrooms, prolonging their shelf-life from 5 days to 12 days [82,83]. More recently, citrus pectin aerogel derived from citrus fruits’ peels has been used for mushroom quality maintenance. Wu et al., developed a composite aerogel using citrus pectin and cellulose nanofibers, featuring a well-preserved fibril network and macropores. This bioaerogel facilitated the gradual release of thymol and maintained relative humidity at around 97% during storage, effectively preserving the postharvest quality and extending the shelf-life of button mushrooms [64]. Further, guar gum (GG), originating from the seed endosperm of *Cyamopsis tetragonolobus*, has been combined with chitosan or sodium alginate to produce active packaging for edible mushroom preservation [51,84].

### 3.2. Animal-Derived Extracts

Compared to plant-derived extracts, there is a limited availability of natural substances of animal origin currently used for food preservation. While chitosan, lactoferrin, and propolis have been widely used in meat and dairy product preservation, chitosan stands out as the primary choice for preserving edible mushrooms [85]. Chitosan, a natural linear polysaccharide with polycationic properties, is obtained by the partial deacetylation of chitin, which is primarily sourced from the crustaceans shells like crabs and shrimps. Its low toxicity, remarkable biodegradability, and regenerative capabilities have garnered significant attention in recent years [86]. Additionally, chitosan has a notable inhibitory effect on the growth of various microorganisms. Due to its outstanding biological activities and film-forming properties, chitosan is considered an ideal biopolymer for producing edible coatings/active packaging films to safeguard mushrooms against quality deterioration. However, a single chitosan coating/film has limitations in its antioxidation, water barrier, and mechanical properties. Thus, chitosan tends to be modified in practical applications to improve the barrier properties and functionality of coatings/films.

The physical modification of chitosan primarily involves simple blending and nano-crystallization. For instance, the combination of chitosan coatings with guar gum significantly inhibited tissue softening and the loss of ascorbic acid and soluble solids in shiitake mushrooms [84]. Zhang et al., Wang et al., and Song et al., blended chitosan with zein to prepare active packaging films containing antibacterial or antioxidant agents, successfully preserving the overall quality of button mushrooms [38,87,88]. Biofilms made from chitosan and dextran have also been shown to delay spoilage time and extend shelf-life up to 28 days at 4 °C [89]. Similarly, Sun et al. utilized a combination of chitosan and hyperbranched Poly-L-lysine to protect oyster mushrooms, markedly reducing membrane damage and enzymatic browning during postharvest storage [90]. Various chitosan-based nanocomposites, such as chitosan/silica and chitosan/titanium, have been developed to enhance the effectiveness of chitosan in mushroom preservation [63,91]. Furthermore, chitosan nanoparticles are regarded as a good delivery system for active substances. Chitosan nanoparticles loaded with *Citrus aurantium* EO effectively enhanced antioxidant properties and prolonged shelf-life up to 15 days [42,92]. On the other hand, graft copolymerization and carboxylation are the main chemical modification approaches used to enhance chitosan for edible mushroom preservation. Previous studies have shown that protocatechuic acid, gallic acid, and caffeic acid have been grafted onto chitosan to develop edible coatings/films. The physico-chemical characteristics, as well as the functionality, of coatings/films are highly dependent on the grafting degree. As reported by Liu et al., protocatechuic acid-grafted chitosan coatings with medium (190.11 mg/g) and high (279.69 mg/g) grafting degrees performed much better than those with a low grafting degree (61.64 mg/g) in the quality maintenance of oyster mushrooms [57]. Both gallic acid-grafted chitosan and acid-grafted chitosan/polylactic acid film packaging have shown promising results in mushroom preservation [59,61]. Additionally, carboxymethyl chitosan, a water-soluble chemical derivative of chitosan, exhibits excellent film/coating forming ability and antibacterial properties. Enoki mushrooms (*Flammulina velutipes*) coated with carboxymethyl chitosan exhibited decreased total viable count, respiration rate, weight loss, and browning degree, as well as increased soluble solid content and sensory appeal, after storage for 12 days, compared to the control group [93].

In addition to chitosan, the utilization of other animal-derived natural products for mushroom preservation is relatively scarce. Li et al. prepared antimicrobial peptide microspheres using cathelicidin-BF (isolated from snake venom) to preserve *Tricholoma matsutake*, extending its postharvest lifespan by up to 20 days [94]. Furthermore, egg white protein, lecithin, and gelatin have been previously employed for edible coatings or active packaging films, showing significant potential in mushroom preservation [50,95].

### 3.3. Microbial-Derived Extracts

Certain microbial metabolites and their derivatives possess inherent antimicrobial capacity. Due to their ability to inhibit the growth of pathogenic or spoilage microorganisms in food products, these substances have potential to serve as natural, safe, and effective bio-preservatives. Bacteriocins and microbial polysaccharides are the primary microbial-derived compounds utilized in mushroom preservation. Bacteriocins, which are low-molecular weight peptides or proteins with antibacterial and antifungal properties, can be synthesized by various microbial species [96]. Nisin, a prominent bacteriocin produced by lactic acid bacteria (LAB), is recommended by FAO as a bacteriostatic agent and is widely used in preserving fruits, vegetables, meats, and dairy products [97]. Nisin can be used alone or in combination with other compounds. When combined with plasticized poly(lacitc acid), nisin effectively preserved the quality attributes and sensory characteristics of *B. edulis* wild edible mushrooms, thereby extending their shelf-life to 18 days [98]. Nisin demonstrates strong inhibitory effects against Gram-positive bacteria, some of which are involved in mushroom spoilage. For example, nisin assisted with lactic acid and ultrasound was effective in controlling the growth of *Listeria monocytogenes* and *Escherichia coli* O157:H7 in enoki mushrooms [99]. To improve its efficacy, nisin is often integrated into edible coatings for mushroom preservation. An edible coating based on sodium alginate enriched with nisin, thyme EO, and L-cysteine significantly delayed the senescence, aerobic mesophilic bacterial growth, and quality deterioration of *Pholiota nameko* mushrooms [100]. Similarly, an edible coating containing nisin, nano-silica, and chitosan proved to be efficient in prolonging the shelf-life of button mushrooms with acceptable quality [91]. Apart from nisin, *ε*-poly-L-lysine (*ε*-PL) has also been utilized in mushroom preservation. As a natural antimicrobial peptide, *ε*-PL exhibits broad-spectrum antibacterial activity against both Gram-positive and Gram-negative bacteria. According to Wei et al., *ε*-PL inhibited the growth of *Lactococcus lactis* in enoki mushrooms and prevented quality degradation during storage [101].

In addition to bacteriocins, microbial polysaccharides have been extensively used for mushroom preservation. Gellan gum, a water-soluble microbial polysaccharide, can serve as a polymer coating. Criado et al. prepared an edible coating using gellan gum loaded with modified cellulose nanocrystals which effectively decreased the respiration rate, delayed ripening, and extended the shelf-life of mushrooms [60,75]. Pullulan, another microbial polysaccharide, possesses unique properties and excellent film-forming capacities, enabling it to inhibit fungal growth. The use of pullulan as a stabilizer in a cinnamaldehyde emulsion coating showed a beneficial impact on reducing the accumulation of ROS in golden needle mushrooms [102]. Furthermore, dextran, an extracellular polysaccharide synthesized by extracellular enzymes of LAB strains, has been studied. Díaz-Montes et al. combined dextran isolated from *Leuconostoc mesenteroides* SF3 with chitosan to create biofilms that extended the spoilage time of mushrooms up to 28 days at 4 °C [89].

### 3.4. Other Natural Extracts

Mushrooms and marine algae are plentiful sources of natural products. They contain a wide range of compounds with antimicrobial/antioxidant properties. The rich diversity of mushrooms and algae offers promising natural substances for food quality maintenance. Nevertheless, current research on bioactive components extracted from mushrooms and marine algae for use in edible mushroom preservation is limited.

Some mushroom polysaccharides have been used to preserve postharvest edible mushrooms. For example, oyster mushrooms coated with *Oudemansiella radicata* water-soluble polysaccharide (ORWP) displayed reduced weight loss and MDA production, as well as increased nutrient contents and antioxidant activities [103]. Furthermore, ORWP significantly inhibited the mycelial growth of *Penicillium digitatum* by disrupting cell membrane integrity. Similarly, ORWP was found to effectively maintain the overall quality of shiitake mushrooms [104]. More interestingly, Guo et al. employed shiitake mushroom polysaccharide for the preservation of postharvest shiitake mushrooms, observing that the polysaccharide film could substantially suppress mushroom browning and softening, while activating a regulatory system to counteract oxidative stress during storage [105]. Additionally, mushrooms are well-known abundant natural sources of ergothioneine (ET), which possesses the potential to delay the quality deterioration of food products owing to its remarkable antioxidant properties [106]. In a storage test involving button mushrooms, a 0.12 mmol/L ET solution effectively inhibited the activities of PPO, POD, PAL, CAT, and mushroom browning by modulating the expression of genes *AbPPO1*, *AbPPO3*, *AbPPO5*, *AbPAL1*, and *AbPAL2* [107].

The abundance and renewability of marine algae make them an inexhaustible natural resource. In recent years, there has been a growing global interest in the application of algae-derived natural products for food preservation and quality improvement [12]. Among these bio-constituents, polysaccharides stand out as one of the most significant in mushroom preservation. Alginate is a natural unbranched linear polysaccharide found abundantly in marine brown algae, such as *Macrocystis aeruginosa* and *Sargassum*. Edible coatings/films based on alginate create an immediate barrier on the surface of fresh mushrooms, preventing water and oxygen filtration and inhibiting microbial infection during postharvest storage, thus preserving their quality [100]. Active agents can be added to alginate-based coatings/films to enhance their efficiency [49,51,95,108]. Additionally, carrageenan, another algae polysaccharide with exceptional gel-forming properties, is utilized for maintaining edible mushroom quality [83].

## 4. Techniques for the Application of Natural Products in Mushroom Preservation

### 4.1. Surface Impregnation

**Figure 3:**
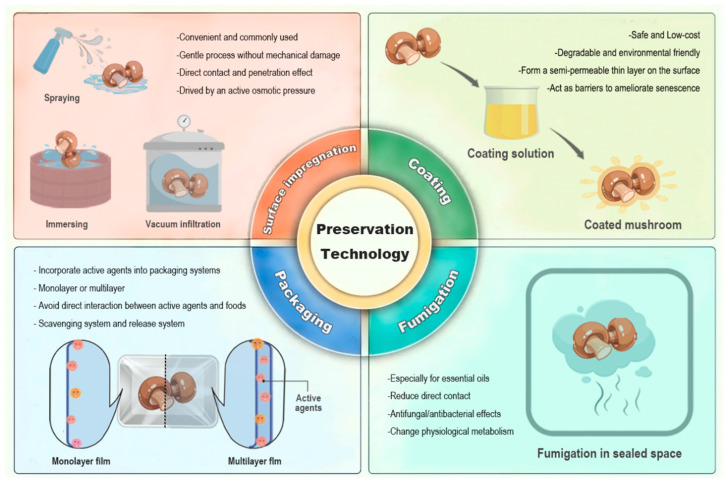
Techniques for the application of natural products in edible mushroom preservation.

The technique of soaking is commonly used to deliver preservatives to edible mushrooms. It involves immersing fresh mushrooms in a solution containing dissolved preservatives for a specific duration, followed by removing and drying (Figure 3). It is a gentle process without mechanical harm, making it particularly suitable for the preservation of edible mushrooms. The natural preservatives present in the solvent permeate the cellular structure of the mushrooms through their surfaces, facilitated by the presence of intercellular spaces that allow for penetration. Soaking occurs through active osmotic pressure rather than external force, driven by the concentration gradient of solutes between the solution and the interior of the cells, facilitating the diffusion and infiltration of preservatives into the mushroom tissues [109].

In comparison to soaking, vacuum infiltration provides a more efficient means of transporting preservatives into tissues within a shorter duration. However, this technique requires large equipment and ample space, making it predominantly suitable for laboratory-scale applications [70]. Conversely, spraying is the most convenient approach. The preservative solution is directly sprayed onto the surface of fresh mushrooms using a spraying device, subsequently permeating the tissues gradually [65]. Owing to its convenience, this method can be used at various postharvest stages, including transportation, storage, and retailing. Nonetheless, it should be noted that the penetration efficacy of the sprayed preservatives is relatively limited, potentially failing to reach the innermost part of tissues, thereby constraining its broad utility. Fundamentally, soaking, vacuum infiltration, and spraying all entail an infiltration process subsequent to direct contact. Table 1 delineates the principal applications of natural products in edible mushroom preservation through surface impregnation.

### 4.2. Fumigation

Fumigation serves as a prevalent method for delivering lipid volatile/gaseous preservatives in agricultural food products’ preservation. In this procedure, mushrooms are enclosed in a sealed environment containing lipid volatile/gaseous preservatives, facilitating their diffusion and penetration into tissues (Figure 3). In comparison to surface impregnation, fumigation has the advantages of straightforward application, high permeability, and not requiring extra equipment or space [113]. Among various natural products, EOs have volatility and are frequently used for preserving mushroom quality by the means of fumigation, such as clove, cinnamaldehyde, thyme, and peppermint oils (Table 1). Presently, EOs are predominantly employed in mushroom preservation as antibacterial/antifungal agents to deter disease progression during storage [32]. While research indicates that spraying EOs is more effective than fumigation in controlling the growth of microorganisms, the high concentration of EOs may have a negative impact on the flavor and odor of agricultural products [109]. By contrast, fumigation minimizes direct contact with mushrooms and leverages their antimicrobial properties, thereby helping to mitigate adverse effects. Moreover, fumigation allows the volatile components of EOs to permeate the interior of mushroom tissues, altering the physiological metabolism of mushrooms during storage, thereby enhancing their resistance to pathogens and activating their defense systems [34].

### 4.3. Edible Coating

Recently, there has been a growing acceptance of edible coatings for food preservation [86]. These coatings typically comprise polysaccharides (chitosan, alginate, cellulose, gums, etc.), proteins, lipids, and additives like antimicrobial, antioxidant, and anti-browning agents, most of which are derived from natural origins. Edible coatings can form a semi-permeable layer on the mushroom surface after dipping, soaking, or spraying. This layer acts as a barrier to reduce solute and moisture transfer, respiration, internal gas exchange, oxidation, and microbial contamination, thereby aiding in maintaining the quality of mushrooms [114] (Figure 3). Compared to traditional packaging, edible coatings are low-cost, convenient, and environmentally friendly [29]. As the raw materials used in edible coatings are biodegradable, there are no concerns regarding the accumulation of residues in food and adverse effects on human health. Over the past few years, a variety of edible coatings have been successfully developed to preserve edible mushrooms and extend their shelf-life. The preservation effects of different edible coatings on mushrooms have been compiled in Table 2. Empirical studies have shown that the application of edible coatings confers significant advantages in terms of regulating postharvest physiological processes (softening, browning, cap opening, respiratory intensity, etc.) and inhibiting the proliferation of various microorganisms including mesophilic, psychrophilic, yeast, molds, and others. Furthermore, these edible coatings exhibit a positive impact on preserving the nutritional value and bioactive components of postharvest mushrooms.

### 4.4. Active Packaging

Packaging plays an important role in the physical preservation of food. Over the last few decades, the function of food packaging has expanded beyond traditional containment and preservation to encompass versatility, including microbiological safety, shelf-life extension, and environmental sustainability [43]. An increasing number of packaging is manufactured with naturally sourced biopolymers, known as bio-packaging. They have been proposed as an alternative to synthetic materials. Active packaging, an emerging technology, incorporates active ingredients (antimicrobial, antifungal, antioxidant, and anti-browning agents, oxygen and ethylene scavengers, etc.) into packaging systems to enhance their protective properties, rather than directly adding them to foods [117]. Thus, this method helps to prevent interactions between active agents and food items [71]. In general, active packaging is categorized into scavenging and release systems based on the functionality of active agents. In scavenging systems, packaging inhibits spoilage by absorbing oxygen, while in release systems, active compounds are released in a controlled manner onto the surfaces of mushrooms. Active packaging can be designed in various ways, such as incorporating active agents into a monolayer film for packaging edible mushrooms or using multilayer films (Figure 3). The barrier layer in a multilayer film prevents the migration of active agents to the outside of package, and the inner layer controls their gradual diffusion [118]. Currently, active packaging is extensively utilized to delay the deterioration and senescence of postharvest mushrooms, including button mushrooms, straw mushrooms, shiitake mushrooms, oyster mushrooms, and white beech mushrooms, as outlined in Table 3.

## 5. Preservation Mechanisms of Natural Products for Postharvest Mushrooms

In recent years, natural products sourced from various origins have been widely utilized to preserve the quality of edible mushrooms during postharvest storage and transportation. Comprehensively considering the existing literature, inhibiting the growth and proliferation of pathogenic microorganisms, enhancing the antioxidant system, and regulating cell wall metabolism are key mechanisms involved in the preservation process. These aspects may operate independently or synergistically to mitigate quality deterioration and extend the shelf-life of mushrooms.

**Figure 4:**
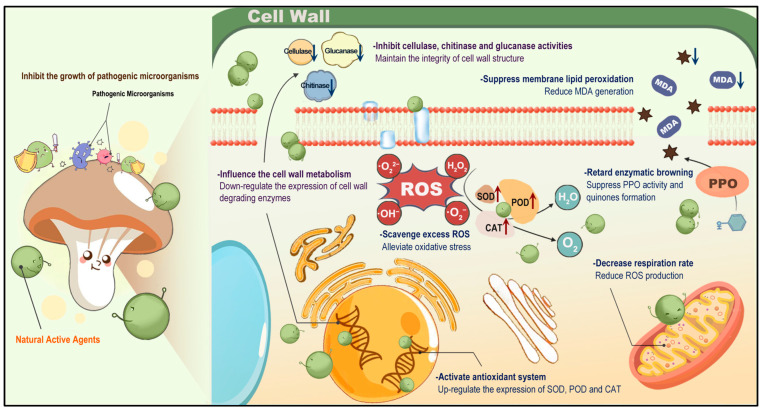
Preservation mechanism of natural products on postharvest edible mushrooms.

Postharvest mushrooms are frequently vulnerable to various pathogenic microorganisms that utilize them as nutrient substrates for growth and proliferation [36]. These microorganisms not only degrade the intracellular matrix but also diminish the central vesicles, leading to cell collapse and mushroom rot. Many natural products have been reported to possess potent antimicrobial capacities or contain antimicrobial components, such as EOs [8,35,38,39,41,49,50,74,81,83,112,116,119], phenolics [58,67], bacteriocins [94,98,99,101], and polysaccharides [103]. They have the potential to inhibit the growth or eradicate microorganisms that adhere to the surface of mushrooms (Figure 4). Their antimicrobial effectiveness varies depending on the specific active ingredient as well as the type of microorganism strain. For example, Gram-negative bacteria are more resistant to plant-derived EOs than Gram-positive bacteria. Natural preservatives act by directly interacting with microorganisms, penetrating and disrupting the integrity of their cell membranes and cell walls, interfering with signal transmissions to the cell membranes, and ultimately inhibiting their reproduction and altering their pathogenicity. This interaction also disrupts the electron transport chain, resulting in protein dysfunction, inactivation of key enzymes, oxidative stress, mitochondrial damage, and DNA damage [26]. Moreover, the growth of microorganisms is dependent on suitable environmental conditions to maintain metabolic activity. Edible coatings or active packaging films containing natural products can effectively reduce the proliferation of microorganisms by interfering with the exchange of materials (water/gas) between microorganisms and the external environment [119].

The decline in mushroom quality under adverse conditions is primarily attributed to the overproduction and accumulation of ROS as a major intrinsic factor, in addition to extrinsic factors. Normally, ROS production and elimination are in dynamic equilibrium, with endogenous antioxidants responsible for counterbalancing the excess free radicals. However, excessive oxidation disrupts electron transport in mitochondria, leading to increased ROS generation and oxidative damage [62]. These free radicals assault biological macromolecules present in mushrooms, such as proteins, lipids, and nucleic acids, triggering enzyme inactivation, DNA damage, membrane lipid peroxidation, and cell structure disruption, ultimately compromising postharvest mushroom quality. The preservation of edible mushrooms by various natural products has been linked to the regulation of redox homeostasis, primarily involving EOs [33,34,35,38,39,43,79,92,100,102,116], phenolics [29,57,59,61,62,65], and polysaccharides [103,104,105,108]. These active ingredients help reduce ROS generation and accumulation by inhibiting the respiration rate and maintain metabolic activity at a relatively low level during postharvest storage. Consequently, membrane lipid peroxidation is reduced and MDA content declines, significantly alleviating the cell membrane damage and delaying the aging process of mushrooms [65]. Moreover, antioxidant enzymes (SOD, CAT, POD, APX, etc.) are crucial in the defense systems of mushrooms and can be influenced by natural active components through the regulation of related genes like SOD, APX, and PAL (Figure 4). Enhanced levels of antioxidant enzymes mitigate oxidative stress by scavenging free radicals. For instance, SOD converts O_2_^·−^ into H_2_O_2_ and oxygen, while CAT and POD can catalyze the decomposition of H_2_O_2_ into water and oxygen. By effectively eliminating free radicals, these enzymes contribute to maintaining the quality of mushrooms [29]. Additionally, natural substances have the potential to suppress the activity of PPO to prevent mushrooms from enzymatic browning by down-regulating the expression of PPO-related genes like *AbPPO1*, *AbPPO3*, and *AbPPO5* [107]. Furthermore, many researches indicate that active ingredients can reduce the loss of phenolic compounds, ascorbic acid, and other endogenous antioxidants, thereby preventing or mitigating damage caused by excessive ROS [81,90].

The utilization of natural products has been shown to effectively slow down the softening of mushrooms and preserve their textural qualities during postharvest storage. This effect is primarily due to the positive impact of natural products on cell wall metabolism, as illustrated in Figure 4. The loss of firmness in mushrooms is attributed to the breakdown of cell wall components by various metabolic enzymes, including chitinases, glucanases, and cellulases. Throughout storage, the activities of these enzymes typically increase, leading to structural changes in the cell wall and mushroom quality deterioration. Active agents have the capability to uphold cell wall integrity and delay softening by inhibiting the activities of enzymes responsible for cell wall degradation, such as *β*-glucanase, *β*-1,3-glucanase, *β*-galactosidase, polygalacturonase, chitinase, and cellulase, among others. In recent years, an expanding body of research has identified several relevant genes like *Leglu1*, *Letlg1*, *Leexg1*, *Leexg2*, *Lechi1*, and *Lepus30a* that play a role in the degradation of mushroom cell walls [22]. Consequently, post-transcriptional regulation appears to be the primary mechanism for reducing the activities of these key enzymes activities and alleviating the softening of postharvest edible mushrooms.

## 6. Conclusions and Future Perspectives

Freshly harvested mushrooms are highly perishable, presenting a significant challenge for their preservation. In recent years, natural products derived from various sources, including plants, animals, microorganisms, and others, have been extensively utilized to preserve edible mushrooms and prolong their shelf-life. These natural substances primarily include essential oils, polyphenols, polysaccharides, bacteriocins, and other bioactive compounds. The preservation mechanisms of these natural substances involve inhibiting the growth of harmful microorganisms, boosting the antioxidant system, and regulating cell wall metabolism. However, there are several aspects deserving further consideration and discussion. While this review has highlighted many natural products, they only represent a fraction of the potential resources available. It is imperative to explore a broader range of natural products derived from different raw materials to evaluate their effectiveness in preservation. Furthermore, certain factors currently limit these products’ commercial applications, such as their stability, cost considerations, and the standardization of potential industrial production processes. Additionally, the integration of innovative natural products with traditional preservatives or preservation techniques remains an underexplored area. Novel combinations and refinements could effectively delay the deterioration of mushroom quality. Future research endeavors should focus on elucidating the targets of bioactive substances and their underlying molecular mechanisms. A comprehensive understanding of the functional mechanisms of these substances is crucial for the development of more efficient preservation strategies.

## Figures and Tables

**Table 1:** Applications of natural products in edible mushroom preservation through surface impregnation and fumigation.

Technique	Natural Products	Concentration	Edible Mushrooms	Preservation Effects	Ref.
Soaking	Glycine betaine	1.0, 2.5, and 4.0 mM	*A*. *bisporus*	Inhibited weight loss, respiration rate, cap opening and browning, maintained high contents of polyphenol and ascorbic acid, protected cell membrane structure, enhanced activities of SOD, POD, and CAT	[110]
Soaking	Brassinolide	1 and 3 μM	*A*. *bisporus*	Restrained browning, inhibited weight loss and electrolyte leakage, reduced ROS accumulation and MDA production	[111]
Soaking	Gallic acid	2.5 mg/mL	*Black truffles*	Reduced microbial contamination induced by *Pseudomonas* spp., *Enterobacteriaceae*, and *Eumycetes*, reduced off-flavors, maintained sensory quality	[58]
Soaking	*Oudemansiella radicata* polysaccharide	5, 10, and 15 g/L	*Pleurotus ostreatus*	Reduced weight loss, electrolyte leakage, and MDA production, enhanced antioxidant enzyme activities, inhibited mycelial growth of *Penicillium digitatum*	[103]
Soaking	Nisin	0.1% (*w*/*w*)	*Flammulina. velutipes*	Inhibited the growth of *Listeria monocytogenes* and *Escherichia coli* O157: H7, exhibited synergistic effects with lactic acid and ultrasound	[99]
Soaking	*ε*-Polylysine and nisin	0.15 and 0.30 g/kg for *ε*-Polylysine; 0.10 and 0.20 g/kg for nisin	*F. velutipes*	Controlled the growth of *Lactococcus lactis*, inhibited weight loss and color-changing, increased soluble solids content, maintained mushroom quality	[101]
Vacuum infiltration	Pistachio green hull extract	0.05% (*w*/*v*)	*A. bisporus*	Inhibited weight loss, browning, and softening, enhanced phenolic content, antioxidant activities, and sensory quality	[70]
Spraying	Ergothioneine	0.12 mM	*A. bisporus*	Delayed browning and softening, reduced MAD production and electrolyte leakage, maintained phenolics and ascorbic acid levels, inhibited PPO, POD, PAL, and CAT activities	[107]
Spraying	Oxyresveratrol and resveratrol	0.03% (*w*/*w*)	*L. edodes*	Alleviated softening, browning, and decay, suppressed oxidation and hydrolysis of membrane phospholipids	[65]
Fumigation	Cinnamaldehyde, clove and thyme oils	1, 5, and 10 μL/L	*A*. *bisporus*	Inhibited browning and cap opening, maintained phenolics and VC contents, decreased PPO and POD activities, increased PAL activity, and reduced microorganism counts	[15]
Fumigation	Cinnamaldehyde, clove and thyme oils	1, 5, and 10 μL/L	*L. edodes*	Increased antioxidant activities of CAT, SOD, APX, and GR, retained the contents of phenolic compounds and flavonoid, retarded mushroom sensory quality deterioration	[33]
Fumigation	Peppermint oil	5, 10, 20, and 50 μL/L	*A*. *bisporus*	Restrained browning, increased contents of phenolic, flavonoid, soluble protein, and total sugar, improved antioxidant system and regulated the related genes, alleviated membrane lipid peroxidation	[34]
Fumigation	Lemongrass, citronella, mint and clove oils	1 and 2 μL/g	*Pleurotus. florida*	Increased total phenol content and the activity of PAL, decreased PPO and POD activities, showed a positive impact on mushroom quality	[35]
Fumigation	Geranium oil and lemongrass oil	60 and 80 μL/L for geranium oil; 40 and 60 μL/L for lemongrass oil	*A*. *bisporus*	Reduced weight loss, browning, softening, and count of fungi, maintained the overall quality of mushrooms	[112]

**Table 2:** Applications of natural products in edible mushroom preservation through edible coatings.

Edible Mushrooms	Edible Coating	Preservation Effects	Ref.
*P. eryngii*	Protocatechuic acid-grafted chitosan coating	Coatings reduced browning, softening, weight loss, respiration rate, MDA content, electrolyte leakage, superoxide anion production, hydrogen peroxide content, and PPO activity, as well as enhanced antioxidant enzyme activities.	[57]
*A. bisporus*	Tragacanth gum coating incorporating 100, 500, and 1000 ppm *Zataria multiflora* Boiss. essential oil	Coated mushrooms maintained 93.47% tissue firmness and showed reduction in microbial counts from yeasts and mold. Coatings decreased browning index, promoted the accumulation of phenolic compounds and ascorbic acid.	[81]
*A. bisporus*	Tragacanth gum coating incorporated with 100, 500, and 1000 ppm *Satureja khuzistanica* essential oil	Coatings decreased softening, browning, and microorganism counts, enhanced the levels of total phenolics and ascorbic acid.	[8]
*L. edodes*	Chitosan (1%) and guar gum (5, 15, and 25%)	Mushrooms coated with 1% chitosan and 15% guar gum maintained tissue firmness, slowed the loss of soluble protein and ascorbic acid.	[84]
*A. bisporus*	*Konjac glucomannan* (0.48%)/carrageenan (0.6%)/nano-SiO_2_ (0.3%) coatings	Coating maintained the color of mushrooms, inhibited respiration rate and the degradation of proteins and polysaccharides, and delayed the senescence process.	[83]
*P. nameko*	Sodium alginate enriched with 1% (*v*/*v*) thyme oil, 0.3 g/L L-cysteine, and 0.4 g/L nisin	Coatings inhibited weight loss, cap opening, browning degree, MDA production, PPO, POD, and cellulase activities, as well as preserved the contents of soluble sugar, ascorbic acid, and soluble proteins.	[100]
*A. bisporus*	Gellan gum edible coating loaded with CNCs and CNCs-g-GA	Coated mushrooms showed low color change, decreased firmness loss, and increased cap diameter.	[60]
*A. bisporus*	Gum-, agar-, sodium alginate-, egg white protein-, and lecithin-based edible coatings	All coatings prevented weight loss, coloring, and browning and suppressed respiration rate and ethylene production.	[95]
*A. bisporus*	Gellan gum edible coating loaded with CNCs (0, 10%, and 20%)	All coatings decreased color change, suppressed respiration rate, and prolonged the shelf-life.	[75]
*L. edodes*	Polysaccharide isolated from *Oudemansiella radicata*	Coated mushrooms had reduced weight loss, improved firmness, reduced browning, decreased MDA content, PPO, POD, protease, cellulase, and chitinase activities, and improved physical microstructure.	[104]
*A. bisporus*	Alginate-based coating containing cinnamaldehyde oil nanoemulsion (0.025%, 0.05%, and 0.1%)	All coatings decreased respiration rate, weight loss, polyphenol oxidase activity, and *Pseudomonas* counts, and increased retention of firmness, color, total polyphenols, and antioxidant activities.	[49]
*A. bisporus*	Titanium dioxide nanoparticles and chitosan with the addition of thymol and tween	Composite coatings enhanced color, reactive oxygen species, and antioxidant activity, while the addition of thymol and tween reduced respiration rates and increased phenolic contents.	[63]
*A. bisporus*	Titanium/chitosan, silica/chitosan	All nanomaterial coatings enhanced antioxidant enzymes’ activities, suppressed respiratory spike onset, and blocked carbon dioxide passage from inside to outside.	[91]
*A. bisporus*	Apple peel powder (1.0 to 1.4%, *w*/*v*), carboxymethyl cellulose (1.2 to 1.8%, *w*/*v*), tartaric acid (0.375%), and glycerol monostearate (1%)	Mushrooms coated at optimum condition (apple peel powder 1.17% wt/vol, carboxymethyl cellulose 1.8%, and dipping time 75 s) maintained a high level of quality parameters, prolonging their shelf-life up to 5 and 9 days under ambient and refrigerated conditions, respectively.	[72]
*A. bisporus*	Pectin-, chitosan-, sodium alginate-, and carboxymethyl cellulose-based edible coatings, individually and/or in combination with N-acetyl cysteine	All coatings delayed weight loss and cap opening, and improved phenolic content and antioxidant activity. Sodium alginate coatings were the most effective, followed by pectin coatings.	[115]
*A. bisporus*	Protocatechuic acid (118 mg/L)-CaCl_2_ (0.83%)-NaCl (0.55%)- pullulan (0.30%) composite edible coatings	Coated mushrooms showed reduced respiration rate, browning degree, MDA content, PPO activity, and increased POD, CAT, PAL, and T-SOD levels and contents of soluble protein and nucleic acid.	[29]
*F. velutipes*	Carboxymethyl chitosan (1%)-based coatings alone or loaded with glutathione (0.2%)	Coated mushrooms exhibited decreased total viable count, respiratory rate, weight loss, and browning degree, and increased soluble solid content and sensory overall likeness.	[93]
*T. matsutake*	Antimicrobial peptides (Cathelicidin-BF-30, 1 g/L) and antimicrobial peptide microspheres (0.5 and 1.0 g/L)	Antimicrobial peptide microspheres coatings maintained the firmness, reduced the loss of ascorbic acid and total sugar, and decreased PPO and POD activities.	[94]
*A. bisporus*	Sodium alginate (2%), ascorbic acid (0.2%), and their combination	The composite edible coatings reduced weight loss and color degradation and inhibited PPO and POD activities.	[108]
*A. bisporus*	Chitosan nanoparticle film loaded with cajuput essential oil	Coatings reduced weight loss and respiration rate, and improved antioxidant activity, maintaining the sensory quality of mushrooms.	[43]
*A. bisporus*	Aloe vera and gelatin edible coatings containing *Shirazi thyme* essential oil nanoemulsion	Coatings maintained physicochemical, microbiological, and sensory properties, and lowered microbial counts including mesophilic bacteria, yeasts, and molds.	[50]
*F. velutipes*	Pullulan (6%)-stabilized soybean phospholipids/cinnamaldehyde emulsion	Emulsion coating inhibited the accumulation of reactive oxygen species, improved the effectiveness of delaying active free radical scavenging enzymes, and significantly prolonged their shelf-life.	[102]
*A. bisporus*	Aloe vera gel-based coating with orange peel essential oil (500 and 1000 μL/L)	Coatings reduced browning, softening, cap opening, weight loss, and respiration rate, retained total phenolics and flavonoids, enhanced antioxidant enzyme activities and sensory evaluation, and inhibited microbial growth.	[116]
*P. ostreatus*	Chitosan and hyperbranched poly-L-lysine composite coatings	Coatings reduced rot degree, weight loss, browning, and MDA content, retained reducing sugar, VC, soluble protein, and total phenolic content, increased CAT, SOD, PAL, and POD activities, and decreased PPO activity.	[90]

**Table 3:** Applications of natural products in edible mushroom preservation via active packaging.

Edible Mushroomds	Active Packaging	Preservation Effects	Ref.
*B. edulis*	Biobased poly(lactic acid) films with 0.5% nisin	Packaged mushrooms showed decreased weight loss, PPO activity, and bacterial counts, increased firmness and total soluble solids, and improved sensory quality. Their shelf-life was extended up to 18 days.	[98]
*A. bisporus*	Biobased poly(lactic acid)/poly(*ε*-caprolactone) blend films with different cinnamaldehyde (0, 3, and 9 wt%)	The biofilms reduced weight loss, softening, coloring, respiration rate, and microbial counts, and retained overall acceptability within limit of marketability.	[119]
*A. bisporus*	Chitosan nanoparticles biopolymer containing *Citrus aurantium* essential oil or cumin oil	Coated mushrooms showed accumulated phenolic compounds and ascorbic acid, increased CAT and SOD activities, decreased PPO activities, and retained overall acceptability of mushrooms.	[42,92]
*A. bisporus*	Bacterial cellulose active films containing pomegranate peel extract, green tea extract, and rosemary extract	These biofilms decreased weight loss, browning, and microbial counts, preserved total phenol and ascorbic acid, and enhanced antioxidant property.	[66]
*A. bisporus*	*Konjac* glucomannan/carrageenan/nano-silica films with nano-silica	The biofilms markedly reduced the browning index, delayed the weight loss and softening, and extended the shelf-life of mushrooms.	[83]
*A. bisporus*	Gallic acid grafted chitosan film	Packaged mushrooms showed low respiration rate, browning degree, MDA content, electrolyte leakage rate, superoxide anion production rate, and hydrogen peroxide content.	[59]
*L. edodes*	Polyvinyl alcohol/cinnamon essential oil/*β*-cyclodextrin (CPVA-CEO-*β*-CD) nanofibrous films	The biofilms improved hardness, maintained better color of mushrooms, and decreased weight loss rate.	[45]
*A. bisporus*	Mesoporous silica nanoparticles/starch-based films loaded with cinnamon essential oil	The biofilms had antimicrobial activity against the CNRMA 03.0371 strain and the FJ09 species commonly found in postharvest white mushrooms.	[78]
*P. ostreatus*	Combination of modified atmosphere packaging with bilayer active packaging consisted of gelatin with pomegranate peel powder coated on the polyethylene film	Packaged mushrooms showed the lowest weight loss, the highest score for the overall acceptability.	[71]
*A. bisporus*	Zein/ethyl cellulose hybrid electrospun nanofibers encapsulated cinnamon essential oil	The biofilms decreased weight loss, and maintained the firmness and overall postharvest quality.	[46]
*A. bisporus*	Chitosan/zein films containing *α*-tocopherol	Packaged mushrooms had lower weight loss, relative leakage rate, browning index, respiration rate, PPO, POD activities, and MDA content, as well as higher firmness, CAT, SOD activities, total phenolic content, and DPPH radical scavenging activity.	[87]
*A. bisporus*	Chitosan and dextran produced by a *Leuconostoc mesenteroides* strain blend films	The biofilms reduced moisture loss, softening, and browning and delayed spoilage time to 28 days at 4 °C.	[89]
*A. bisporus*	Starch–cinnamon essential oil microencapsulated in bioactive paper	This paper enhanced the stability of internal environment, reduced respiration and microbial infection, inhibited cell membrane damage.	[41]
*A. bisporus*	Chitosan/zein complicated film loaded with lemon essential oil	The biofilms suppressed PPO and POD activities, inhibited the growth of microorganism and mushroom browning, decreased respiration rate, and enhanced antioxidant capacity and texture properties.	[38]
*A. bisporus*	Poly(lactic acid)/corn starch/eucalyptus/eucalyptus leaf essential oil (15 mL/100 mL) microencapsulated active bilayer degradable film	The biofilms significantly inhibited the decomposition of soluble proteins and respiration rate, reduced moisture loss, maintained the white color, delayed aging, and effectively extended shelf-life.	[52]
*Volvariella volvacea*	Polylactic acid/poly (butylene adipate-co-terephthalate)/thermoplastic starch loaded with clove oil and peppermint oil	The biofilms reduced PPO activity, maintained the content of TPC, inhibited mushroom autolysis, and extended the shelf-life of mushrooms.	[117]
*A. bisporus*	Nanocellulose/nanohemicellulose (1%) loaded starch nanocomposite (0.25%, 0.5%, 0.75% and 1%) packaging	Packaged mushrooms exhibited better retention in pH, color, and firmness and retained better quality of mushrooms.	[76]
*A. bisporus*	Caffeic acid-grafted chitosan/polylactic acid films	The biofilms delayed browning and respiration rate, decreased the accumulation of MDA, superoxide radicals, and hydrogen peroxide, and enhanced the activities of SOD and CAT in mushrooms.	[61]
*A. bisporus*	Citrus pectin aerogel fortified with cellulose nanofibers	Hardness, color, total phenol content, cell membrane integrity, and total antioxidant capacity were maintained and fresh-keeping period was extended to 5 days.	[64]
*A. bisporus*	Caffeic acid-grafted chitosan/polylactic acid film packaging	Packaged mushrooms had a more complete structure, a higher content of phospholipids, stronger activities of GPAT and SMS, and lower activities of LOX, PLC, and PLD.	[62]
*A. bisporus*	Polylactic acid and mesoporus silica nanoparticles loaded with clove essential oil	Packaged mushrooms showed low weight loss, high total phenolics and ascorbic acid contents, reduced growth of microorganisms, and down-regulated PPO and POD activities.	[39]
*A. bisporus*	Glutenin/tamarind gum-based active film loaded with binary microemulsion of melatonin/pummelo essential oil	The biofilms enhanced antioxidation, microorganism inhibition, and free-radical-scavenging properties, effectively delaying the senescence of postharvest mushrooms.	[53]
*L. edodes*	Gelatin- and shiitake stalk polysaccharide (1%, 1.5%, and 2%)-based film	The biofilms reduced MDA production, increased the activity of resistance-related enzymes, and regulated the activities of PPO, tyrosinase, PAL, and *β*-1,3 glucanase.	[105]
*L. edodes*	Polylactic acid (PLA)/cellulose extracted from water bamboo bio-based antibacterial packaging material loaded with cinnamaldehyde	The biofilms inhibited the mycelial growth and spore germination of *Aspergillus niger* and *Trichoderma harzianum* isolated from shiitake mushroom, and improved the storage quality.	[74]
*A. bisporus*	A low-temperature plasma-modified film, loaded with cinnamon essential oil in mesoporous silica nanoparticles (Santa Barbara Amorphous-15)	The biofilms maintained whiteness, water content, and hardness, reduced respiration rate and MDA content, and retained higher key enzyme activity of the phenylpropane metabolic pathway.	[79]
*A. bisporus*	Chitosan/zein/cinnamon essential oil (0.6%) sustained-release active film	Packaged mushrooms showed lower weight loss and spoilage index, higher firmness, and better appearance.	[88]
*A. bisporus*	Sodium alginate/guar gum-based nanocomposite film incorporating a *β*-Cyclodextrin/persimmon pectin-stabilized baobab seed oil Pickering emulsion	The biofilms decreased browning degree and shrinkage, and maintained a satisfactory visual quality during the storage process.	[51]
*H. marmoreus*	Cinnamon–clove compound essential oil microcapsules/graphene oxide/polyvinyl alcohol/polylactic acid composite films	The biofilms maintained the firmness, total soluble solids, proteins, and ascorbic acid contents and inhibited mushroom browning and respiration rate, prolonging the shelf-life for 4 days at 4 °C.	[47]

## Data Availability

The original contributions presented in the study are included in the article, further inquiries can be directed to the corresponding author.
